# Editor’s Book Table

**Published:** 1870-06

**Authors:** 


### Editor's Book Table.

*The Indigestions: or Diseases of the Digestive Organs, Functionally Treated.* By THOMAS KING CHAMBERS, Honorary Physician to H. R. H. the Prince of Wales, etc., etc. Third American Edition, revised. Philadelphia: Henry C. Lea. 1870. Pp. 383.

It is a noticeable fact that a third edition not being yet required in England, the author sends the MS. for publication to America. We fancy that this is due to the fact that Dr. C. is progressive to an extent more pleasing to the American than the British mind. He has a philosophical and at the same time an intensely practical method which has for many years especially won our esteem and confidence, as we have again and again indicated through the *Journal.* This treatise is worth a whole shelf-full of systematic treatises that could be selected even among modern works.

Indigestion complicates about all ailments. Oftentimes it is all there is of the so-called disease. No matter what the nomenclature is, modern physiology teaches that the point of prime interest is to take care of nutrition. Appropriate local conditions being secured so far as possible, the physician's business then is to furnish suitable nutriment on the one side and due elimination on the other. These three are all. What medicines can do, may be more or less questionable, but about these things there can be no question.

Some ten dozen cases are added to those contained in the first edition. There are ten chapters and a copious index. He discusses, after the introduction: Indigestions, acute and chronic: Indigestions of various Foods; Local Pains in the Stomach arising from Indigestions; Vomiting; Flatulence; Diarrhœa; Constipation and Costiveness; Nerve Disorders connected with Indigestions; Causes of the Indigestions. Starting from a physiological basis he illustrates departures from the normal condition by an immense number of cases clearly and concisely stated. Treatment is given, with a perspicuity and definiteness of purpose, which will be found peculiarly gratifying to the practitioner. To those physicians who rarely buy a book, and look more to what they call practical matters than theories, we would say, you will make money by purchasing this book, reading and pondering it well. The influence its author has already had on the professional work of the time has been very great, and it is certain to increase the more he is studied, and the more he writes.

*The Membrana Tympani, in Health and Disease.* By Dr. A. Politzer, of Vienna. Translated by A. Mathewson, M. D., and G. H. Newton, M. D., Surgeons of the Brooklyn Eye and Ear Hospital. Pp. 183. For sale by Messrs. Keen & Cooke, 113 & 115 State St., Chicago.

The student of aural medicine can form no more excellent habit than that of examining with care the external meatus and membrana tympani. Whatever may be said of the value of other means of diagnosis in diseases of the ear, a careful inspection of the meatus and membrane reveals by far most frequently the conditions upon which pain and impaired hearing depend.

It would be difficult to estimate the amount of physical pain and mental distress caused by neglected diseases of the ear, which could be easily prevented, if physicians were in the habit of simply making an examination of these portions of the ear, by means of concentrated light, thrown into the external meatus with the concave mirror, as recommended by V. Troetsch.

The brief but valuable monograph of Politzer has been placed within the reach of every American student by the excellent translation of Drs. Mathewson and Newton.

The work is an admirable statement of what is known concerning the membrane tympani and its diseases.

The value of the work is much enhanced by numerous chromo-lithographs, illustrating the normal and the abnormal conditions of the membrane.

The translators merit the thanks of the profession for this valuable labor. E. L. H.

*The Gentleman's Stable Guide: Containing a Familiar Description of the American Stable; the most approved method of Feeding, Grooming, and General Management of Horses; together with Directions for the care of Carriages, Harness, etc.* By ROBERT MCCLURE, M. D. V. S., author of Diseases in the American Stable, Field and Farm Yard. Philadelphia: Porter & Coates, 822 Chesnut Street. Pp. 184. 12mo.

The character of this book is well indicated in the title. Every physician who keeps a horse will find herein many valuable hints upon the various topics included. It is a very sensible and practical treatise. The hygienic care of horses is very clearly set forth, and the therapeutics sufficiently simple and "common sense" in their character.

### Books Received.

*Obstetric Operations, Including the Treatment of Hemorrhage.* By ROBERT BARNES, M. D., Lond., F. R. C. P., Obstetric Physician to, and Lecturer on Midwifery and the Diseases of Women and Children at St. Thomas' Hospital, etc., etc. With Additions, by BENJAMIN F. DAWSON, M. D., late Lecturer on Uterine Pathology in the Medical Department of the University of New York, etc., etc. New York: D. Appleton & Company, 90, 92 and 94 Grand street. 1870. 8 vo, pp. 483.

*Anatomy, Descriptive and Surgical.* By HENRY GRAY, F. R. S., etc., etc. Fifth Edition, revised and enlarged. Philadelphia: Henry C. Lea. 1870.

*The Private Life of Galileo.* Compiled principally from his Correspondence and that of his eldest daughter, SISTER MARIA CELESTE, Nun in the Franciscan Convent of St. Matthew in Arcetri. Boston: Nichols & Noyes. 1870. Pp. 300.

*Only a Girl: Or, a Physician for the Soul.* A Romance from the German of Wilhelmine Von Hillem. By Mrs. A. L. Wister. Philadelphia: J. B. Lippincott & Co. 1870.

*A Practical and Systematic Treatise on Fractures and Dislocations.* By A. Jackson Howe, M. D., Professor of Anatomy in the Eclectic Medical Institute. Cincinnati: Charles F. Wilstach & Co. 1870. Pp. 424.

*United States Sanitary Commission Memoirs.* Surgical. Cambridge: Riverside Press. 1870.

*Basham on the Kidneys.*

The late period at which the above were received, prevents further notice in the present number.

---

### Pamphlets.

*Reduction of Dislocations.* By William Warren Green, M. D., Professor of Surgery in the Medical School of Maine.

Prof. Green has issued a pamphlet of eleven pages upon the above named subject, being a reprint from the *Boston Medical and Surgical Journal.* He tells us in a prefatory note that the paper was commenced two years previously to October last; and, in some additional remarks, that it was completed previous to the publication of Prof. Bigelow's book on the Hip.

Prof. Green believes that the untorn portion of the ligament or ligaments constitutes the main impediment to the reduction of all dislocations. He tells us that he sat under the surgical instruction of Prof. Moses Gunn in the winter of 1854–5, and that the principle which that teacher then applied to two luxations of the hip, (viz., the dorsal and ischiatic), and to the several dislocations of the shoulder, was so convincing to him that he adopted it and has since then extended its application to all luxations. This general application of the principle, Prof. Green ably supports by both argument and reference to cases occurring in his practice.

*Archives of Ophthalmology and Otology.* Edited and Published simultaneously in English and German. By Prof. H. KNAPP, M. D., in New York, and Prof. S. Moos, M. D. in Heidelberg. Vol. 1, No. 1. New York: William Wood & Co. Carlsruhe: Chr. Fr. Muller'sche Hoffbuchhandlung. 1869.

It is the intention of the editors to issue the "Archives half yearly, in spring and autumn," by separate independent numbers, each to contain about 250 to 300 pages, and two numbers to form a volume. The present No. contains 364 pages, besides ten or twelve leaves of plates and diagrams. It is well printed upon beautiful paper, and the chromo-lithographic plates, as promised in the prospectus, are superb. The matter is entirely original, and contributed by men of eminence both in this country and Europe. If the present No. is any indication, the "Archives" will prove invaluable, not only to the ophthalmologist and otologist but to the general practitioner. Address Wm. Wood & Co., publishers, New York City. $7.00 per annum.

